# Prognostic value of regional metastasis in squamous cell carcinoma of the tongue and floor of mouth

**DOI:** 10.5935/1808-8694.20130134

**Published:** 2015-10-08

**Authors:** Ali Amar, Abrão Rapoport, Otávio Alberto Curioni, Rogério Aparecido Dedivitis, Claudio Roberto Cernea, Lenine Garcia Brandão

**Affiliations:** a PhD in Otorhinolaryngology and Head and Neck Surgery, Federal University of São Paulo, Brazil (Assistant Professor, Department of Head and Neck Surgery and Otorhinolaryngology, Heliópolis Hospital, São Paulo, Brazil); b Associate Professor, Department of Surgery, School of Medicine of the University of São Paulo, Brazil (Technical Director, Department of Health, Heliópolis Hospital, São Paulo; Surgeon, São José Hospital, São Paulo, Brazil); c Associate Professor, School of Medical Sciences in Santos, UNILUS, Santos, Brazil (Head of the Department of Head and Neck Surgery and Otorhinolaryngology, Heliópolis Hospital, São Paulo; Surgeon, São José Hospital, São Paulo, Brazil); d Associate Professor; Supervisor of the Larynx Group, Department of Head and Neck Surgery and Otorhinolaryngology, University Hospital of the School of Medicine, University of São Paulo, Brazil (M.D.); e Associate Professor, Department of Head and Neck Surgery, School of Medicine, University of São Paulo, Brazil (Associate Professor, Department of Head and Neck Surgery, School of Medicine, University of São Paulo, Brazil); f Full Professor, Department of Head and Neck Surgery, School of Medicine, University of São Paulo, Brazil (Full Professor, Department of Head and Neck Surgery, School of Medicine, University of São Paulo, Brazil). Department of Head and Neck Surgery and Otorhinolaryngology, Heliópolis Hospital, São Paulo - SP, Brazil

**Keywords:** carcinoma, squamous cell, lymphatic metastasis, mouth neoplasms

## Abstract

The presence of metastatic nodes is a survival-limiting factor for patients with mouth tumors.

**Objective:**

To evaluate the causes of treatment failure in carcinomas of the tongue and floor of the

mouth due to staging.

**Method:**

This study included 365 patients with squamous cell carcinoma of the mouth treated from 1978 to 2007; 48 were staged as T1, 156 as T2, 98 as T3, and 63 as T4, of which 193 were pNo and 172 pN+.

**Results:**

Among the pN+ cases, 17/46 (36.9%) of the patients not treated with radiation therapy had relapsing tumors, against 46/126 (36.5 %) of the patients who underwent radiation therapy. Success rates in the group of subjects submitted to salvage procedures were 16/51 (31.3%) for pN0 patients and 3/77 (3.9%) for pN+ patients.

**Conclusion:**

Salvage procedure success and survival rates are lower for pN+ patients; pN+ individuals also have more relapsing local disease.

## INTRODUCTION

Squamous cell carcinoma of the tongue is the most common tumor of the oral cavity, accounting for 25% to 40% of the cases of mouth neoplasms. The oral tongue's rich lymphatic network and musculature make it the site in the mouth most frequently associated with neck metastasis^1^.

Local recurrence still ranks as the most common shortcoming in the treatment of carcinomas of the mouth. However, the presence of metastatic nodes before the start of treatment is a major adverse prognostic factor, and cuts by approximately 50% the patient's expected survival2., 3.. Recurrences are common, especially among patients with advanced disease - stages III and IV - and within the first two years after treatment. Despite the poor prognosis associated with metastatic disease, regional recurrence is uncommon in carcinomas of the upper aerodigestive tract^4^.

This study aimed to determine the causes of failure in the treatment of carcinomas of the tongue and floor of mouth due to staging.

## METHOD

This study was approved by the Research Ethics Committee of the institution where it was held and given permit No. 071/2000.

Patient charts of patients with squamous cell carcinoma of the tongue and floor of the mouth treated with surgery between January 1978 and December 2007 in a reference tertiary care center were reviewed. Patients with asymptomatic disease and individuals followed for less than 12 months were excluded. A total of 365 patients were selected. In patients with stage I disease not submitted to elective neck dissection, only the second episode of recurrence occurred after neck surgery was considered as regional recurrence. Disease in another anatomic site or distant at least two centimeters from the original tumor was diagnosed as second primary tumor. Patients submitted to salvage procedures were deemed successfully treated when no evidence of new relapsing disease was found in their latest visit to the hospital. All patients were restaged according to the 2002 TNM staging system. The number of episodes of local, regional, and distant relapsing disease and second primary tumors were assessed based on primary tumor site, staging, and postoperative radiation therapy. Regional recurrence and the distance to the second primary tumor were considered separately.

Actuarial survival rates were calculated using the Kaplan-Meier curve and differences between groups by the log-rank test. The chi-square test was used for qualitative variables. Multivariate analysis used logistic regression. Statistical significance was attributed to differences with *p* < 0.05.

## RESULTS

The floor of the mouth was the site of the primary tumor in 198 cases, followed by the tongue in 160 patients, and both sites in seven individuals. Patient mean age was 53 years (22 to 84 years). Forty-eight tumors were staged as T1; 156 were T2 tumors; 98 were T3; and 63 were T4. In terms of metastasis, 193 patients were pN0 and 172 were pN+. The types of recurrence are described in Tables 1 and 2. Postoperative radiation therapy was offered to 27 pN0 patients and 126 pN+ patients. Local recurrence was seen in 17/46 (36.9%) pN+ patients not treated with radiation therapy and in 46/126 (36.5%) pN+ patients submitted to postoperative radiation therapy (*p* = 0.95).

**Table 1:** Types of relapsing disease per pN stage.

Recurrence	pN0 n = 193	pN+ n = 172
None	104	58
LR	28	43
RR	13	17
DR	4	11
LR + RR	10	17
LR + DR	1	3
LR + RR + DR	3	0
RR + DR	0	3
Second tumor	33	21

^*^ 3 pN0 patients and 1 pN+ with a second tumor had had locoregional recurrence. LR: Local recurrence; RR: Regional recurrence; DR: Distance recurrence.

**Table 2:** Local (LR), regional (RR) and distance (RD) recurrence per pN stage.

	pN0 n = 193	pN+ n = 172	
LR	42 (21%)	63 (36%)	*p* = 0.002
RR	26 (13%)	37 (21%)	*p* = 0.05
DR	8 (4%)	14 (8%)	*p* = 0.10

**Figure 1:**
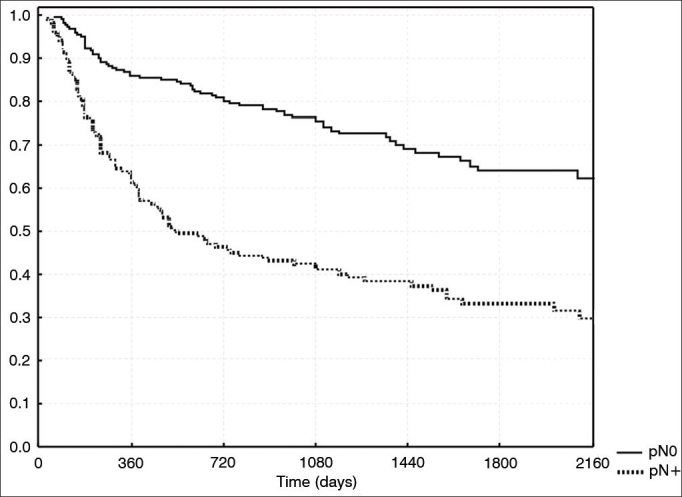
Survival free of disease per pN stage.

Salvage procedures in cases of locoregional recurrence were successful in 16/51 (31.3%) pN0 patients and in 3/77 (3.9%) pN + subjects (*p* = 0.0001). Likewise, surgery for a second primary tumor was successful in 13/29 (44.8%) salvageable pN0 patients and in 2/21 (9.9%) pN+ subjects (*p* = 0.01). Tumor recurrences according to T stage and multivariate analysis are shown in Table 3. In multivariate analysis, factors T and pN had the strongest correlations with local recurrence, with *p*-values of 0.0002 and 0.02, respectively. Actuarial survival according to pN staging is shown in Figure 1.

**Table 3:** Local recurrence per T stage *(p* = 0.0002).

T stage	Local recurrence	Total
T1	5 (10%)	48
T2	37 (23%)	156
T3	35 (35%)	98
T4	28 (44%)	63

## DISCUSSION

Recurrence is a significant prognostic factor; even among patients undergoing additional procedures with curative intent, rates of managed disease are low. Physiologically, recurrence revolves around two possibilities: tumor persistence, in which the disease remains quiescent for a long period of time, or the appearance of a second tumor. Late recurrence seems to be more related to patient characteristics than to the effect of treatment^4^.

The treatment for tumors of the floor of the mouth remains primarily surgical; adjuvant radiation therapy is offered in cases of advanced stage disease or to patients at a high risk of locoregional treatment failure^1^. Despite the lower survival of patients with metastatic nodes, regional control is achieved in most patients submitted to neck dissection and postoperative radiation therapy. The main problem occurs in attaining local control of the disease in more advanced cases as per the T and pN staging systems, as seen in multivariate analysis. Half of the patients with regional recurrence had associated relapsing disease in the site of the primary tumor. The primary tumors of patients with the phenotype for metastatic nodes appear to be more locally aggressive, but recurrence in sublingual or mesiolingual lymph nodes left behind after neck dissection, which could be confused with recurrence at the site of the primary tumor, should also be considered5., 6..

Another factor to impact survival was the lower rate of success of salvage procedures in patients with locoregional recurrences initially staged as pN+, possibly due to the presence of more disease and the greater use of postoperative radiation therapy in such cases^7^. Distant recurrence was probably underdiagnosed, as indicated by the low incidence of reported cases and the fact that additional diagnostic tests were not ordered for patients with unresectable locoregional tumors. Distant metastases are directly related to locoregional disease staging and the presence of recurrence^8^.

Despite the higher incidence of a second tumor in pN0 patients - possibly explained by the longer survival of subjects in this group and the consistent incidence rates of a second tumor with time - treatment success rates were higher for this group of individuals4., 7.. We believe that the greater number of second tumors in subjects without regional metastasis (pN0) may be explained by the natural barrier inherent to the history of oral neoplasms that prevent tumors from spreading to regional nodes. In the absence of lymph nodes, the second tumor occurs in the pharynx, esophagus, lungs or pancreas. The appearance of tumors in the pancreas is more frequent among long-term survivors. Regional metastases are consistent with more aggressive tumors in all involved sites and marked decreases in survival, despite the fact that regional control can be achieved in most cases3., 4., 9..

It has been demonstrated that in patients with HPV-positive tumors, variables associated with regional metastases play a much less important role in prognosis than in patients with HPV-negative tumors. Better survival among HPV-positive patients is due in part to improved locoregional control, presumably reflecting the disease's greater sensitivity to radiation therapy and radiosensitization by cisplatin^10^.

## CONCLUSION

Salvage procedure success and survival rates are lower for pN+ patients when compared to pN0 patients. Individuals staged as pN+ also have more relapsing local disease.
